# HU to RGB transformation with automatic windows selection for intracranial hemorrhage classification using ncCT

**DOI:** 10.1371/journal.pone.0327871

**Published:** 2025-08-06

**Authors:** Dittapong Songsaeng, Akara Supratak, Pantid Chantangphol, Saowapot Sarumpakul, Natsuda Kaothanthong

**Affiliations:** 1 Department of Radiology, Faculty of Medicine Siriraj Hospital, Mahidol University, Bangkok, Thailand; 2 Faculty of Information and Communication Technology, Mahidol University, NakhonPathom, Thailand; 3 Sirindhorn International Institute of Technology, Thammasat University, Pathum Thani, Thailand; 4 Artificial Intelligence Association Thailand, Pathumthani, Thailand; UCSF: University of California San Francisco, UNITED STATES OF AMERICA

## Abstract

This work focuses on preprocessing for classifying five categories of Intracranial Hemorrhage (ICH) using non-contrast computed tomography (ncCT). It involves assigning suitable values to window-width (WW) and window-level (WL) parameters to map Hounsfield Units on ncCT to compatible color components like RGB for display. However, clear visualization is hindered by brain component variations, individual patient conditions, and time elapsed since stroke onset. This paper introduces a preprocessing technique called *HU to RGB Transformation (HRT)*, aimed at enhancing the visualization of hemorrhage on ncCT scans. HRT dynamically selects optimal WW and WL values from predefined settings to accentuate hemorrhage visibility. Furthermore, it leverages multiple brain components, including cerebrospinal fluid and white-and-gray matter, to further refine the delineation of hemorrhagic regions. Experimental results from a deep neural network-based image classification model are utilized to evaluate the effectiveness of the proposed method. This method, serving as an image preprocessing step, demonstrates remarkable capability in classifying five distinct types of Intracranial Hemorrhage and normal slice, achieving an average sensitivity of 89.35% and an average specificity of 96.03%. Moreover, direct assessment of HRT preprocessed images leads to enhanced type classification accuracy by residents, with a sensitivity of 97.39% and a specificity of 96.19%. These results surpass those obtained from reading DICOM files achieving 93.31% sensitivity and 94.81% specificity.

## 1 Introduction

Intracranial hemorrhage (ICH) is a life-threatening medical condition that indicates bleeding within the skull. The compression of blood in the brain can lead to other causes of serious complications and death if proper treatment has not been taken promptly. ICH has five categories: Intraventricular (IVH), Intraparenchymal (IPH), Subarachnoid (SAH), Epidural (EDH), and Subdural (SDH); where each has different causes, symptoms, and treatments[1]. Multiple bleeding location, as well as cerebrospinal fluid (CSF) and gray/white matters, are essential for managing hemorrhage and treatment plan [2, 3].

Non-contrast Computed Tomography scan (ncCT) is an imaging to localize bleeding in brain. Each pixel on a CT slice is represented using a value called Hounsfield unit (HU) [4]. HU was commonly used by radiologists to stratify brain components. To exhibit on the display, two-parameter settings that are window-width (WW) for defining the interested range and window-level (WL) for selecting a middle value of the range are employed to map HU to an image intensity [0,255]. Different settings allow different brain components to be displayed. For example, (WW, WL) for visualizing the brain is (80, 40), subdural is (200, 80), and bone is (2800,600) [5]. However, the stage/or process of disease and disease pathology impact HU and radiologists’ image interpretation. The study of Kamalina *et al* [6] reported that the suitable HU for displaying white matter was 25HU and gray matter was approximately around 35HU with the standard deviation of ± 10–20%. Segawa *et al* [7] found that the white matter enhancement averagely around 24±4HU and the gray matter was 19± 4HU. In the report of Kim *et al* [8], the HU of CSF was 0 to 15, the normal cerebral tissue was less than 40, and the hemorrhage cells were less than 80. The classification in early stroke stage on ncCT in [9] reported that multiple settings (WW, WL) to display brain infarct affect the performance. Although a transformation of HU values to an image intensity could be done [10–13], a manual adjustment prohibits an automatic system. Additionally, the similarity between HU of CSF and small parenchymal hematoma, early hemorrhagic changes in cerebral contusion, and early hemorrhagic transformation of acute infarction necessitates a contrast injection for precise diagnosis by medical practitioners.

Deep learning (DL) is a machine learning approach that extracts knowledge from input data and applies it to infer new information, particularly in medical image interpretation [14–19]. Automated systems employ deep neural networks and brain CT scans with Hounsfield Unit (HU) values to develop predictive models, aiding radiologists in identifying normal studies [20–22]. Various preprocessing techniques, such as image segmentation and color mapping methods, have been proposed to enhance classification performance. Image segmentation methods like thresholding and bounding box techniques have been utilized for classifying hemorrhage regions and assessing brain injuries in Aneurysmal Subarachnoid Hemorrhage patients [23, 24]. Additionally, segmentation has been employed to approximate CSF boundaries using active contour methods [25]. Other approaches involve clustering similar HU values to distinguish clotted and unclotted areas or applying thresholds to separate image regions [26–28]. In tandem with segmentation, color mapping has been introduced to highlight specific body tissues and assign labels for brain regions [29]. However, conventional color mapping techniques only display one specific region per image, whereas multiple categories of Intracranial Hemorrhage (ICH) necessitate the simultaneous presentation of CSF, bone, and white-and-gray matter in non-contrast CT scans [30, 31]. Furthermore, varying shading is essential to depict different hemorrhage conditions.

Radiologists’ review strategy often involves employing multiple window settings to map Hounsfield Unit (HU) values in brain CT scans, aiming to highlight various intensity ranges and detect subtle abnormalities. In line with this approach, several studies have utilized predefined window settings to transform brain CT scans, stacking the resulting images as temporal inputs for deep neural networks to develop Intracranial Hemorrhage (ICH) classification models [32–34]. Commonly, three settings are utilized to eliminate bone interference and depict subdural and brain components [32, 33], whereas ten settings are employed to represent diverse subdural damage and tissue conditions [34]. Using multiple windows simultaneously in radiological imaging can obscure details and introduce conflicting information. Each window setting highlights different aspects of the image, potentially leading to misinterpretation.

This work present a brain ncCT preprocessing method called *HU-RGB Transfomation (HRT) with multiple windows and multiple components* for the ICH classification task. In clinical practice, a radiologist typically relies on multiple window settings to discern variations in brain tissue, by comparing and contrasting different configurations. The proposed HRT method mirrors this approach through the application of various window settings, enabling it to detect changes of boundary points extracted from specific regions and consequently detect the changes to select the most appropriate settings for analysis. This process involves the adaptive assignment of predefined window settings, which allows for the automatic selection of the most suitable configuration for each brain CT scan. This innovative approach not only mirrors the decision-making process of radiologists but also enhances the efficiency and accuracy of classification models in identifying ICH.

**Fig 1:**
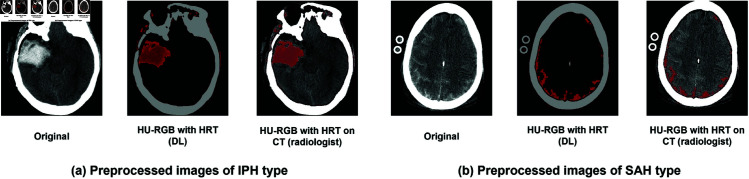
Example of the preprocesssed image of IPH (a) and SAH (b) that will be used as an input for a deep neural network (DL) and review by the radiologists (radiologist).

The proposed preprocessing method offers the capability to identify small parenchymal hematomas, early hemorrhagic changes in contusions, and the initial stages of hemorrhagic transformation in acute infarctions, all while circumventing the need for unnecessary contrast injection. The HRT algorithm intelligently allocates the red color component to the identified hemorrhagic regions based on the Hounsfield Unit (HU) values observed in ncCT. It is used as an image preprocessing for hi-lighting hemorrhages in the brain CT for a classification model using a deep neural network (DNN) to categorize IVH, IPH, SAH, EDH, SDH, and normal brain. Examples of preprocessed images of IPH and SAH types to be used by DNN are shown in Fig 1 (DL). The hi-lighted hemorrhage is aligned on the original brain CT for assisting an types inspection by a radiologist as depicted in Fig 1 (radiologist).

## 2 Related works

### 2.1 Mapping Hounsfield Unit values to an image

In brain CT, Hounsfield Unit (HU) represents the value of each pixel, where the value is related to the density of tissues with the different amounts of X-ray absorbency. The value in each pixel on CT image is from -1000 to 1000. Each pixel in a digital image can be represented using a color space such as RGB, CMYK, or HSV. RGB color is commonly used in many applications, where each pixel combines an intensity of red (R), green (G), and blue (B) color from 0 to 255. Since the range value of HU is wider, visualizing CT on an image requires a transformation function to map the value [–1000,1000] to [0,255].

Windowing is a function that maps a HU value to an intensity value of the interested range with a predefined value of window level (*L*) and window width (*W*). The traditional windowing function specified the range of the given window level *L* and window width *W* as $\left[L-\frac{W}{2},L+\frac{W}{2}\right]$ in order to map to the intensity range [0,255]. The HU value of the pixel *x* is denoted by *h*_*x*_. If the pixels of HU values below $\left(L-\frac{w}{2}\right)$, then the mapped value is *f*_*trd*_(*x*) = 0. The one above $\left(L+\frac{w}{2}\right)$ is *f*_*trd*_(*x*) = 255. Though the transformation function can display the CT image, the pixels that are subtle cannot be displayed effectively [35].

**Fig 2:**
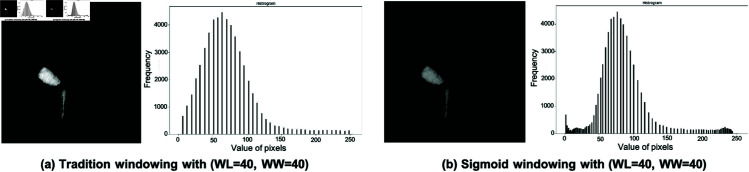
A HU mapped result and a histogram of a tradition widowing method (a) and a Sigmoid function method (b).

The HU values on the CT image of brain tissue are homogeneous and the structure of each tissue is connected, therefore, it is difficult to separate the adjacent tissues due to the small intensity changes and smooth boundaries between the tissues. Instead of assigning a transformed intensity value equal to 0 to the pixels with the HU values outside the interested setting (WW, WL), we would like to assign a constant value. In this way, the sigmoid function is employed [36] to assign the value to the pixels of HU values below $\left(L-\frac{w}{2}\right)$. The equation below transforms each pixel with an HU value equal to *x* into an intensity value

$$f_{sig}(x)=\frac{U}{1+\exp(-(\frac{2}{h}\log(\frac{U}{\epsilon }-1)+\frac{-2l}{w}\log(\frac{U}{\epsilon }-1)))},$$ (1)

where *h* in the Eq 1 is the value of the hounsfield unit. The value of *w* is the window width and *l* is the window level, *U* is the upper limit constant of windowing functions, ϵ is the margin between the upper/lower limits. The function *f*_*sig*_ maps HU values below $\left(L-\frac{w}{2}\right)$ to ϵ, and the ones above $\left(L+\frac{w}{2}\right)$ to *u*. Fig 2 depicts the mapped image using the two mapping methods. For example, the pixels with 85–87 HU in Fig 2(a) were mapped to the intensity value of 255, while Fig 2(b) were respectively mapped to 215, 225, and 233. Although the intensity difference is subtle for humans, the computer system can interpret the changes for the result of the Sigmoid function.

### 2.2 ICH classification

Several studies have utilized predefined window settings to map non-contrast CT (ncCT) slices into images as part of image preprocessing. Commonly used window settings include subdural (WW = 200, WL = 100), bone (WW = 2800, WL = 600), and brain (WW = 80, WL = 40) windows [32, 33]. Additional settings like min-subdural (WW = 130, WL = 50), mid-subdural (WW = 215, WL = 75), and max-subdural (WW = 300, WL = 100) have been employed to highlight additional hemorrhage components [34].

The preprocessed images were stacked and used as input for a temporal series to develop Intracranial Hemorrhage (ICH) classification models using deep neural networks. Sage *et al* proposed a double-branch convolutional neural network based on ResNet-50 architecture, combined with support vector machine and random forest , named DBRF. This model utilized nine preprocessed images for ICH classification [32]. Wang *et al*., winners of the RSNA challenge in 2021, developed Deep Algo, a model designed to mimic radiologists’ scan examination process [33]. Each slice was preprocessed using three predefined window settings. Rajagopal *et al*. applied ten transformed images as input to ten input layers of the Convolutional Long Short-Term Memory model for ICH Detection [34]

## 3 Method

The proposed method called HU-RGB with adaptive window and multiple components (HRT) maps HU value on an ncCT slice to RGB color components. HRT autonomously selects the appropriate (WW, WL) settings for hemorrhage region extraction by converting HU to RGB using eleven pre-defined configurations. Subsequently, the most suitable window is determined based on the largest boundary of the bleeding area identified through active contouring among the transformed images. Additionally, other components such as skull, calcium, and white/gray matter are detected using the specified window setting. To display the clotted/unclotted blood in the hemorrhage regions, we proposed a method that adaptively assign the red color component with respect to HU values on ncCT.

**Fig 3:**
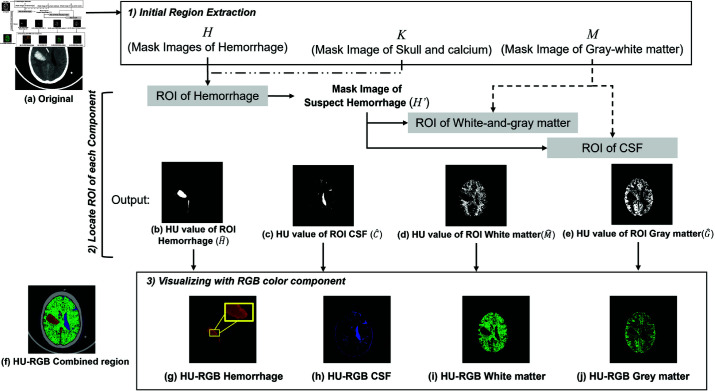
(a)–(e) shows ROI of each component obtained from the proposed HRT method. (a) An original CT, (b) Image of hemorrhage region $I_{\hat{H}}$, (c) Image of CSF $I_{\hat{C}}$, (d) Image of white matter $I_{\hat{M}}$, and (e) Image of gray matter $I_{\hat{G}}$. (f) - (j) depicts the result of the RGB mapped result usnig our proposed method.

Our proposed HRT method comprises three main steps, outlined in Fig 3. Firstly, initial extraction of regions containing skull and calcium, white/gray matter, and hemorrhage is conducted using predefined window settings, detailed in Sect 3.1. Subsequently, region of interest (ROI) detection utilizes these initial regions of multiple brain components to achieve precise delineation, as described in Sect 3.2. Finally, the visualization of CSF and white-and-gray matters employs a fixed RGB color value, while the red component of the hemorrhage ROI adapts to the HU values, as elaborated in Sect 3.3.

### 3.1 An initial region extraction using multiple window settings

Normally, setting for visualizing stroke is WW=80 and WL=40, but the range is too wide such that many HU values are mapped to the same image intensity. Also, suitable values for mapping blood varied to the time after stroke onset such as (WW=130, WL=50), (WW=215, W75), and (WW=300, WL=100) [34]. Visualizing unclotted blood in acute to subacute stages needs a lower HU and a chronic stage requires a higher HU. To find a precise setting of each ncCT, multiple windows WW of 10 and eleven settings of WL are defined for finding an initial hemorrhage region, which is {30,35,40,45,50,55,60,65,70,75}. These predefined values are regarding HU that displayed unclotted and clotted blood studied in [37, 38]. Given a setting, a CT slice is transformed into a gray-scale image using the Sigmoid function. Then, a binary image (denoted by *H*) is generated. The pixels which are mapped to an image intensity value higher than 128 are masked as 1. The mask image obtained from each window setting *l* is defined as *H*_*l*_.

For white/gray matter component and skull, the settings (80,40) and (100,135) are employed respectively. The binary images *K* represents the skull and calcium in the brain, and *M* represents white-and-gray matter. They can be defined analogously as the initial regions of hemorrhage with the same threshold of 128.

### 3.2 A region of interest (ROI) detection using multiple windows and multiple brain components

The aim of the second step is to define ROI of each brain component using the mask image of the other components because the connectivity of each tissue makes the separation of the two components difficult. For example, the HU values of blood and CSF are slightly different. However, the bleeding usually occurs in the area bounded by white-and-gray matter; but the CSF area will not overlap with either hemorrhage or white-and-gray matter regions. The initial regions on the mask images *H*, *K*, and *M* respectively for hemorrhage, skull and calcium, and white-and-gray matter are employed. The ROI of the hemorrhage component applies to the initial region of the skull. While the ROI of CSF and white/gray matter employs the ROI of hemorrhage.

#### 3.2.1 ROI of hemorrhage component.

To ensure the hemorrhage lies within the skull and to avoid the inclusion of calcium in brain, multiple mask images of *H* and *K* are applied.

For each mask image of an initial hemorrhage region *H*_*l*_, let ${\hat{H}}_{l}$ be an n×m matrix of suspected hemorrhage region where the value of each ${\hat{H}}_{l}(i,j)$ is a HU value on the CT image *P*. A mask image of the hemorrhage region is denoted by $H^{\prime }$. If the pixel at the same position on *H*_*l*_ and *K* are masked as 1, that pixel is excluded from the suspected hemorrhage region by assigning ${\hat{H}}_{l}(i,j)=0$. Otherwise, the HU value is assigned to ${\hat{H}}_{l}(i,j)$ with Eq 2 shown below:

$${\hat{H}}_{l}(i,j)=|K(i,j)-H_{l}(i,j)|\times f(P(i,j)),$$ (2)

where f(P(i,j))=P(i,j) for P(i,j)≤110 and 0 otherwise. This work applied 110 as a threshold because the HU higher than 110 is not hemorrhage. The suspect region of hemorrhage is computed for every initial area in *H*_*l*_.

In the Hounsfield unit (HU) scale, -1000 HU to 0 HU represent air or liquid, and 30 HU to 1000 HU indicate soft tissue to calcium. Utilizing boundary points of regions extracted from consecutive window settings starting from low to high HU values, significant decreases effectively highlight hemorrhage regions due to the lower HU values and larger area of liquid in brain.

**Fig 4:**
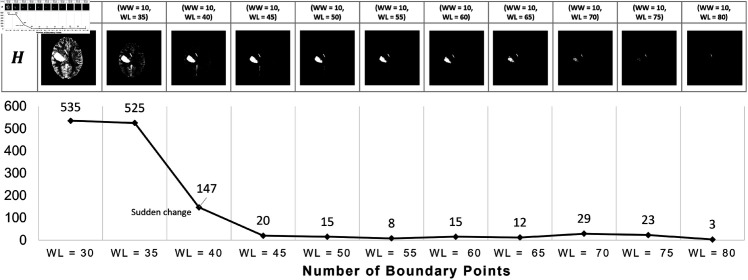
(Top) ROI of suspect hemorrhage images $\hat{H}$ using multiple window level setting in the set *R.* (Bottom) A graph shows the changes of the boundary points extracted from the two consecutive binary images of the setting *R*_*s*_ and *R*_*s* + 1_.

To select ROI of hemorrhage, the boundary of the largest area in each ${\hat{H}}_{l}$ is found by an active contour method. The drastic change in the number of boundary points from the two consecutive windows ${\hat{H}}_{l}$ and ${\hat{H}}_{l+1}$ is tracked. The ROI of hemorrhage is ${\hat{H}}_{l+1}$ which achieves the largest change in the number of boundary points. Fig 4 (Bottom) shows the number of boundary points extracted from each suspect region ${\hat{H}}_{l}$. A pixel on the mask image of the suspect hemorrhage region denoted by $H^{\prime }$ is masked with 1 if the HU value $\hat{H}(i,j)>0$.

#### 3.2.2 ROI of CSF component.

The CSF region is subtle to blood and other liquids in the brain. In this way, the mask images of white-and-gray matter regions (*M*) together with the mask image of the suspect hemorrhage regions ($H^{\prime }$) are utilized. Let $\hat{C}$ be an n×m matrix of the suspected CSF region where each pixel $\hat{C}(i,j)$ is a HU value on the CT image *P*. If the pixels M(i,j) and $H^{\prime }(i,j)$ are masked as 1, the pixel at position (*i*,*j*) is excluded from the suspect CSF by assigning $\hat{C}(i,j)=0$. Otherwise, the HU value is assigned. It is computed with the following equation:

$$\hat{C}(i,j)=|M(i,j)-H^{\prime }(i,j)|\times f(P(i,j)),$$ (3)

where f(P(i,j))=P(i,j) for P(i,j)≤15 and 0 otherwise. The maximum of 15 HU is specified according to the report in [8].

#### 3.2.3 ROI of white/gray matter components.

The mask images of white-and-gray matter *M* and suspect hemorrhage $H^{\prime }$ are used. The range of HU values [*a*,*b*] from the suspect hemorrhage region $\hat{H}$ is defined as *a* = 15 and $b=\min(\hat{H})$. The minimum HU is set to 15 because the average range of 19±4 HU [7]. Let $\hat{M}$ be a region of suspect white matter region where each pixel $\hat{M}(i,j)$ is a HU value on the CT image *P* assigned according to the following equation:

$$\hat{M}(i,j)=|M(i,j)-H^{\prime }(i,j)|\times f(P(i,j)),$$ (4)

where f(P(i,j))=P(i,j) for a≤P(i,j)≤b/2 and 0 otherwise.

The region of the gray matter can be computed similarly to the white matter using different interested ranges of HU value. Let $\hat{G}$ be a binary image of suspect gray matter region where each pixel $\hat{G}(i,j)$ obtained from the following equation:

$$\hat{G}(i,j)=|M(i,j)-H(i,j)|\times f(P(i,j)),$$ (5)

where f(P(i,j))=P(i,j) for b/2<P(i,j)≤b and 0 otherwise.

### 3.3 Visualizing with RGB color component

**Fig 5:**
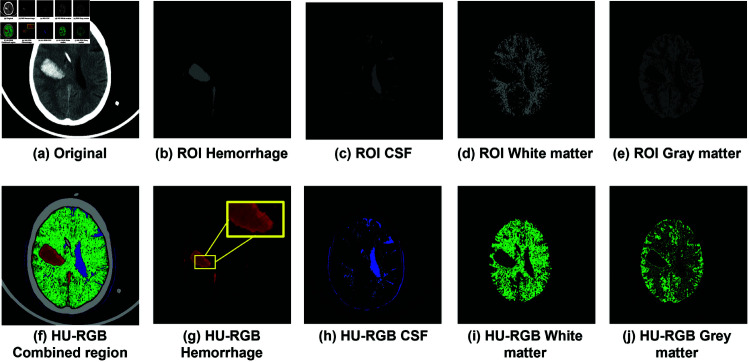
(a)–(e) shows ROI of each component obtained from the proposed HRT method. (a) An original CT, (b) Image of hemorrhage region $I_{\hat{H}}$, (c) Image of CSF $I_{\hat{C}}$, (d) Image of white matter $I_{\hat{M}}$, and (e) Image of gray matter $I_{\hat{G}}$. (f) - (j) depicts the result of the RGB mapped result usnig our proposed method.

ROI of skull and CT artifacts in *K* is mapped to (155,155,155) RGB color component. The non-zero pixels of $\hat{M}$ and $\hat{G}$ showing white-and-gray matter are visualized with the RGB color (0,145,0). Each pixel in $\hat{C}$ of suspect CSF region maps with the RGB color (25,25,145). Examples of the mapped image with RGB color component are shown in Fig 5(g)–5(i).

For hemorrhage, the bleeding and coagulation of the region are differentiated with the red component of RGB. The red value is adaptively assigned according to the HU value on the CT slice *P*. The HU values of hemorrhage ROI, $\hat{H}$, and its mask ${\hat{H}}^{\prime }$ are used. The red component of each pixel is assigned with the RGB component (*r*,0,0) where the value of *r* is between 55 and 255. The lowest value is set to separate the dark red from the black color. The value *r* is defined as follows:

$$r={\hat{H}}^{\prime }(i,j)\left\|255-\left\{\frac{200}{\left(110-\min(\hat{H})\right)}\times (P(i,j)-\min(\hat{H}))\right\}\right\|,$$ (6)

where $min(\hat{H})$ is the minimum HU on the suspect hemorrhage.

**Fig 6:**
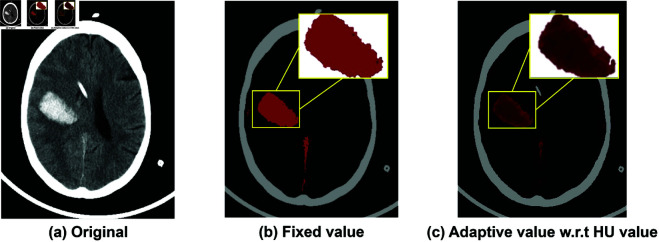
Comparison of a static red color component for visualizing hemorrhage (b) and an adaptive assignment of red color (c).

Example of the adaptive red color of hemorrhage is shown in Fig 5(g). A comparison in Fig 6 exhibits a different shade of blood by assigning darker and brighter shade of red in Fig 6(c), while Fig 6(b) only represent the coverage area of bleeding. The shade of blood enhance a capability to identify small parenchymal hematomas, early hemorrhagic changes in contusions, and the initial stages of hemorrhagic transformation.

### 3.4 Preprocessing ncCT slice using HRT

Our proposed method transforms (HU) on non-contrast CT (ncCT) slices into RGB color components for each brain element. This technique is particularly useful for highlighting bleeding, essential for Intracerebral Hemorrhage (ICH) classification, which encompasses Intraventricular (IVH), Intraparenchymal (IPH), Subarachnoid (SAH), Epidural (EDH), Subdural (SDH), and normal brain cases.

**Fig 7:**
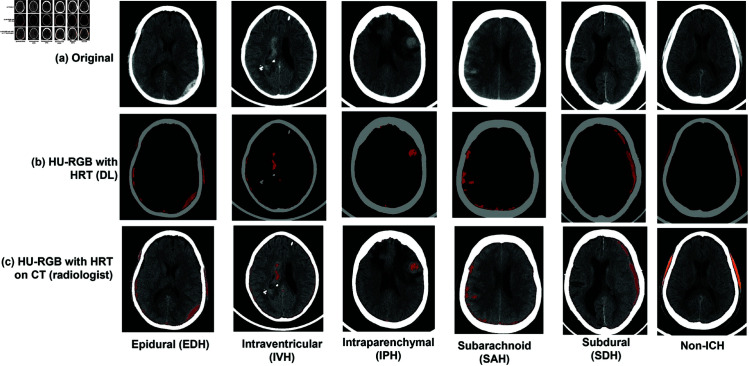
(a) Original images. (b) Preprocessed images of hemorrhage area overlaid with skull. (c) Preprocessed images of hemorrhage area overlaid on brain.

We apply HRT method to generate two styles of preprocessed ncCT slices. To distinguish between ICH types, the location of bleeding relative to the skull is crucial, especially for differentiating IVH and SAH from the rest, as they occur inside the brain. Consequently, only the skull and HRT of hemorrhage regions are displayed on the input image, as illustrated in Fig 7(b). This CT preprocessing facilitates a deep neural network for learning the location and types of bleeding.

Furthermore, to aid radiologists in identifying bleeding, HRT of hemorrhage regions is aligned on the ncCT, as shown in Fig 7(c). This alignment enables radiologists to pinpoint bleeding with cerebral contusion or an early hemorrhagic transformation directly on the original ncCT scan, enhancing diagnostic accuracy.

## 4 Experiment

### 4.1 Data preparation

#### Dataset.

A dataset obtained from an RSNA Intracranial Hemorrhage Detection Challenge dataset [5]. It is a CT brain dataset obtained from multi-institutional, multinational brain hemorrhage CT dataset, and various CT machines. RSNA dataset branch-1 train and branch-1 test were used to train and test the classification models to evaluate the performance of our proposed method. There are 752,803 CT images obtained from multi-institutional, and multinational brain hemorrhage. Multiple labels were assigned to each slice including ICH, non-ICH, and the five hemorrhage types. For each ICH slice, there can be more than one ICH type; therefore, the number of ICH slices for binary classification is lower. The number of images used to develop each model is listed in the original images column in Table 1. For a binary classification, there are 107,933 with ICH and 644,870 with normal brain. For multi-class classification, some slices may be labeled with more than one ICH type. There are 3,145 images with EDH, 47,166 with SDH, 35,675 with SAH, and 36,118 with IPH.

**Table 1:** Number of label images of original images (Ori. Img.), augmented images (Aug. Img.), total images used in the texperiment (Total Img.), training dataset, and testing dataset.

Dataset	Label	Ori. Img.	Aug. Img.	Total Img.	Training	Validation	Testing
RSNA	ICH	107,933	64,040	171,973	128,588	32,555	10,830
	non-ICH	644,870	5	644,875	461,601	115,559	67,715
**Total**		752,803	64,045	816,848	590,189	148,114	78,545
RSNA	EDH	3,145	40,752	43,897	34,704	8,809	384
	SDH	47,166	11,081	58,247	42,902	10,675	4,670
	SAH	35,675	12,077	47,752	35,396	8,803	3,553
	IPH	36,118	13,097	49,215	36,506	9,155	3,554
	IVH	26,205	21,780	47,985	36,157	9,389	2,439
	non-ICH	96,572	1	96,573	23,080	5,778	67,715
**Total**		244,881	98,788	343,669	208,745	52,609	82,315

CQ500 dataset is used as an external validation for evaluating the models developed from the RSNA dataset. The brain CTs of CQ500 were taked from various machine i.e. GE BrightSpeed, GE Discovery CT750 HD, GE LightSpeed, GE Optima CT660, Philips MX 16- slice, Philips Access-32 CT. The label has been considered for each scan. In other words, no label was assigned for each slice. The number of scans with ICH labels are 739 and 530 for non-ICH. For five types, there are 22 scans for EDH, 112 for SDH, 132 for SAH, 368 for IPH, and 74 for IVH.

#### Image preprocessing.

**Fig 8:**
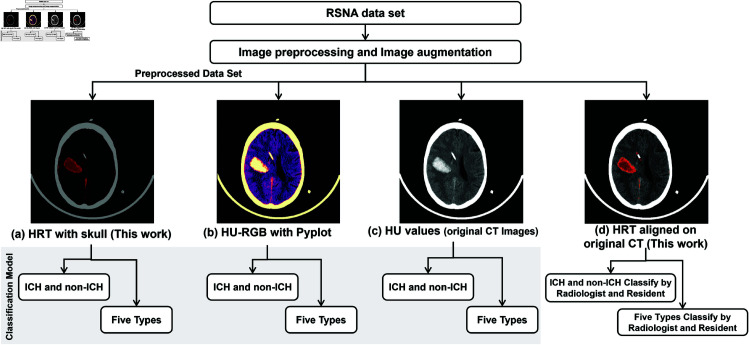
Overview of the experiment to compare the effect of HU-RGB transformation to the classification result. (a) A image using our proposed HRT. (b) Mapped image using Pyplot library. (c) An image of HU value mapping using WW=40 and WL=40. (d) Hemorrhage component of our proposed HRT method aligned on CT image .

Each original ncCT slice is preprocessed using four different methods of HU transformation directly without applying any prior processes. The results of each preprocessing method are depicted in Fig 8(a)–8(d) and applied separately to compare an effect of the preprocessing method towards the classification performance. The detail of each method is described below:

- Our proposed HRT method find the bleeding on each slice and transform HU to RGB. The transformed hemorrhage area and the bone depicted in the same image as shown in Fig 8(a).
- Pyplt is an available library for HU-RGB mapping. This work, the window setting the setting value (40,40) is applied for mapping as shown in Fig 8(b)
- HU values are mapped to an intensity value with the window setting value (40,40) as shown in Fig 8(c).
- The hemorrhage area extracted by the proposed HRT method is aligned on an original CT image as shown in Fig 8(d) for a radiologist and a residence to determine the scan.

#### Image augmentation and dataset division.

Due to an unbalance of the slices, the images for a binary classification and a multi-class classification are augmented separately for each task. An augmentation was applied by randomly selecting CT slices of each preprocessing method to apply an augmentation function arbitrarily. Four augmentation functions are considered: 1) flip vertically, 2) randomly rotate between -15 and 15 degrees, 3) re-scale using the scale from 0.8 to 1.2 of the original image, and 4) crop the slice from the center point using a Python library [39]. The number of augmented images on each label and the total number from images employed in the experiment can be found in the columns *Aug. Img.* and *Total Img.*, respectively in Table 1. The ICH images for a binary classification task are augmented and the total images becomes 171,973. The augmented images of each ICH type for a multi-class classification task is varied to the original image. For example, the original and augmented images of EDH type is 43,897 and SDH is 58,247 images. The augmented image of each label from the RSNA dataset was separated into training and testing sets as shown in the columns *Training* and *Testing*. The training dataset of label ICH and non-ICH is for developing a binary classification model and the five types are for multi-class classification. The validation dataset is used during the model training with the deep neural networks, while the testing dataset is for evaluating each model.

### 4.2 ICH classification models and evaluation metrics

Two classification models are presented. The first model is a binary classification for separating a slice with ICH, while the second one is for classifying types of ICH. The binary classification applied ICH and non-ICH labels, while the multi-class applied five ICH types. Only the slice that has been classified as ICH by the first model is employed by the second model to categorize into one of the five types. The classification result of the second model is a set of five probability values, where each corresponds to types of EDH, SDH, SAH, PH, and IVH, respectively. The type that achieves the highest probability is a classification result of the input.

#### Deep neural network model (DNN).

**Fig 9:**
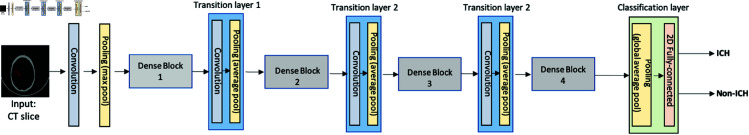
The overview proposed model architecture for classifying ICH and non-ICH on each slice.

**Fig 10:**
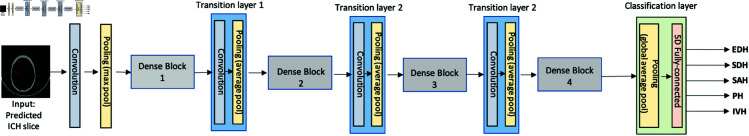
The overview proposed model architecture for classifying five types of ICH on each slice.

The two models integrate a pre-trained DenseNet-121 architecture [40] for training. Given a CT slice, the first model as shown in Fig 9 uses a 2D fully-connected layer as the last layer for classifying ICH or non-ICH. A categorical cross-entropy loss is employed for parameter tuning. DenseNet-121 is also used for separating the five types of ICH. A 5D fully connected layer is employed in the classification layer. See Fig 10, for illustration. The model was trained with a learning rate of 0.001, Softmax activation function, RMSprop optimizer, sparse categorical cross-entropy loss, and 1000 epochs. The same parameter settings were used for training the five-type classification model with a learning rate of 3e-4, a Sigmoid activation function, and binary cross entropy loss. The experiments were conducted on the Intel Xeon Platinum 8280 Processor and the graphic unit interface of the NVIDIA Quadro RTX 8000 machine.

The model trained with our proposed HRT shown in Fig 8(a) is denoted by *HRT-DNN* and the Pyplot in Fig 8(b) is called *Pyplt-DNN*. Lastly, the model trained with the fixed window for mapping in Fig 8(c) is represented as *HU-DNN*.

#### Evaluation metrics.

The classification performance was measured using accuracy (Acc.), sensitivity (Sens.), and specificity (Spec.). The accuracy shows the overall performance of the model in predicting both positive and negative classes. The sensitivity measures the correctness of the model predicting positive classes while the specificity measures negative classes.

### 4.3 Experimental result

Six experiments were conducted to assess the effectiveness of our proposed method. These included binary classification, multi-class classification, and classification by both radiologists and residents. Additionally, we compared the utility of our preprocessing method in clinical routines with other methods. Furthermore, we demonstrated the adaptation of HRT preprocessing with MRI using the BraTS dataset. Finally, we conducted an ablation study to investigate the effect of assigning a threshold of 110HU during hemorrhage ROI detection.

#### 4.3.1 Binary classification.

We conducted experiments to compare the classification performance of our proposed HRT preprocessing technique with Pyplt transformation and HU values. The preprocessed images with HRT also compared with ResNet50 and EfficientNet-B0. Furthermore, the experiments of Deep Algo [33] and DBRF [32] were conducted using the same training, testing, and validation sets of the RSNA dataset for the classification of ICH and non-ICH slices. The data distribution can be found in Table 1.

**Fig 11:**
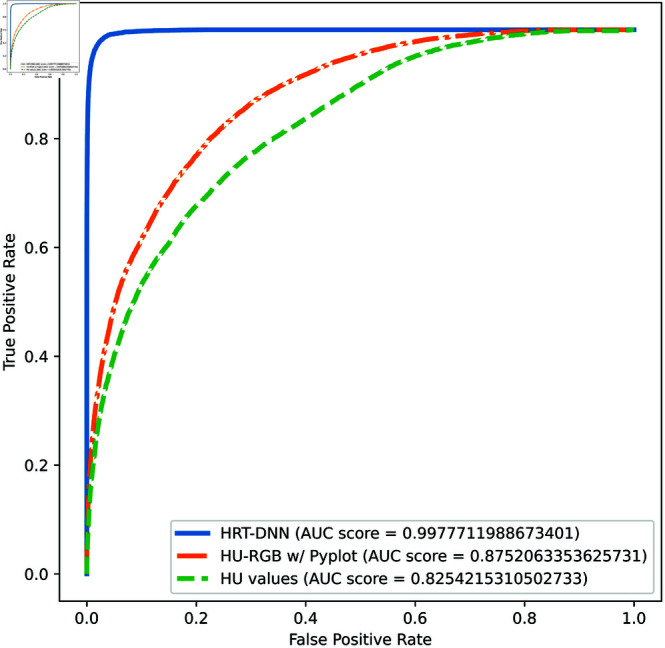
Comparison of ROC of binary classification models of HRT-DNN (our method), Pyplot-DNN, and HU-DNN using RSNA dataset.

Table 2 shows that the classification performance of our proposed HRT preprocessing using the deep neural network (HRT-DNN) achieved an average sensitivity of 95.10%, specificity of 98.93%, and accuracy of 98.40%. The model trained using Pyplt preprocessed images (Pyplt-DNN) achieved an average sensitivity of 64.75%, specificity of 43.77%, and accuracy of 88.96%, which was lower. The model trained with HU values mapped to intensity levels (HU-DNN) achieved an average sensitivity, specificity, and accuracy of 18.39%, 99.04%, and 87.92%, respectively. The AUC of the three methods for the binary classification tested with RSNA was 0.9978 with HRT-DNN, 0.8254 with HU-DNN, and 0.8752 with Pyplot-DNN. The ROC curve is depicted in Fig 11.

**Table 2:** Binary classification performance of the proposed HU-RGB with adaptive window preprocessing compared with the other methods using RSNA and CQ500 datasets.

Dataset	Predict Label	Method	Acc.	Sens.	Spec.	AUC
RSNA	ICH/non-ICH	HRT-DNN	98.40%	95.10%	98.93%	0.9978
		Pyplt-DNN	88.96%	64.75%	43.77%	0.8752
		HU-DNN	87.92%	18.39%	99.04%	0.8254
		HRT-ResNet	86.11%	83.89%	86.47%	0.9082
		HRT-EfficientNet	91.92%	87.32%	92.65%	0.9486
		Deep Algo [33]	97.24%	93.67%	97.81%	0.9917
		DBRF [32]	92.13%	90.45%	92.39%	0.9552
CQ500	ICH/non-ICH	HRT-DNN	92.67%	89.31%	97.36%	0.9443
		Pyplt-DNN	60.84%	64.28%	56.03%	0.6067
		HU-DNN	50.67%	53.99%	46.04%	0.5116

In addition to the result of HRT-DNN that utilized DenseNet121, the preprocessed images were trained with ResNet50 (HRT-ResNet) and EfficientNet-B0 (HRT-EfficientNet). The HRT-EfficientNet achieved 91.92% accuracy, 87.32% sensitivity, 92.65% specificity, and AUC of 0.9486; while the HRT-ResNet obtained 86.11%, 83.89%, 86.47%, and 0.9082, respectively. Compared to Pyply-DNN and HU-DNN, the images that employed our proposed HRT techniques had better performance.

The accuracy of HRT-DNN for a binary classification was also higher than the previous works that stacked the images transformed using multiple window settings input to deep neural work called Deep Algo [33] and DBRF [32] as shown in Table 3. The Deep Algo achieved 97.24% accuracy that is slightly lower than our HRT-DNN, while DBRF obtained 92.13% accuracy.

**Table 3:** Overall performance of multi-class classification using RSNA and CQ500 datasets.

Dataset	Predict Label	Method	Acc.	Sens.	Spec.	AUC
RSNA	Five types ICH	HRT-DNN	99.59%	91.48%	99.92%	0.9994
		Pyplt-DNN	97.34%	57.38%	98.72%	0.9647
		HU-DNN	97.00%	26.09%	99.77%	0.9271
		HRT-ResNet	88.52%	80.93%	88.80%	0.9099
		HRT-EfficientNet	93.18%	85.29%	93.45%	0.9632
		Deep Algo [33]	98.42%	89.87%	98.73%	0.9864
		DBRF [32]	92.91%	89.87%	98.73%	0.9638
CQ500	Five types ICH	HRT-DNN	95.76%	80.24%	97.26%	0.9118
		Pyplt-DNN	85.58%	54.73%	89.32%	0.7291
		HU-DNN	68.67%	25.42%	73.93%	0.4811

The CQ500 dataset was used as an external validation to test the model trained with RSNA, our proposed method HRT-DNN achieved the highest sensitivity of 89.31%, specificity of 97.35%, and accuracy of 92.67%. The other methods achieved lower sensitivity, specificity, and accuracy of 64.28%, 56.03%, and 60.84%, respectively for the Pyplt-DNN model and 53.99%, 46.04%, and 50.67% for HU-DNN.

#### 4.3.2 Multi-class classification.

The overall performance for a multi-class classification can be found in Table 3. For the model trained with RSNA dataset for multi-class classification, our proposed HRT-DNN method achieved an average sensitivity of 91.48%, specificity of 99.92%, and accuracy of 99.59%. The other methods achieved sensitivity, specificity, and accuracy of 57.38%, 98.72%, and 97.34% respectively for Pyplt-DNN and 26.09%, 99.77%, and 97.00% for HU-DNN. Using HRT with ResNet achieved 88.52% accuracy and HRT-EfficientNet obtained 93.18%. Though the accuracy of the models using our proposed HRT is lower, the sensitivity is much higher. The sensitivity of HRT-ResNet and HRT-EfficientNet is 80.93% and 85.29% respectively; while Pyply-DNN and HU-DNN achieved 57.38% and 26.09%. Compared to the previous work Deep Algo has 98.42% accuracy and DBRT has 92.91% accuracy with the sensitivity of 89.87% sensitivity, which is lower than our proposed model.

Testing the multi-class classification model with the CQ500 dataset, HRT-DNN achieved the overall performance shown in Table 3 of 80.24%, 92.26%, and 95.76% sensitivity, specificity, and accuracy, respectively. The results of the other two models were lower with a sensitivity of 54.73%, specificity of 89.32%, and accuracy of 85.58% for Pyplt-DNN and 25.42%, 73.93%, and 68.67% respectively for HU-DNN.

**Fig 12:**
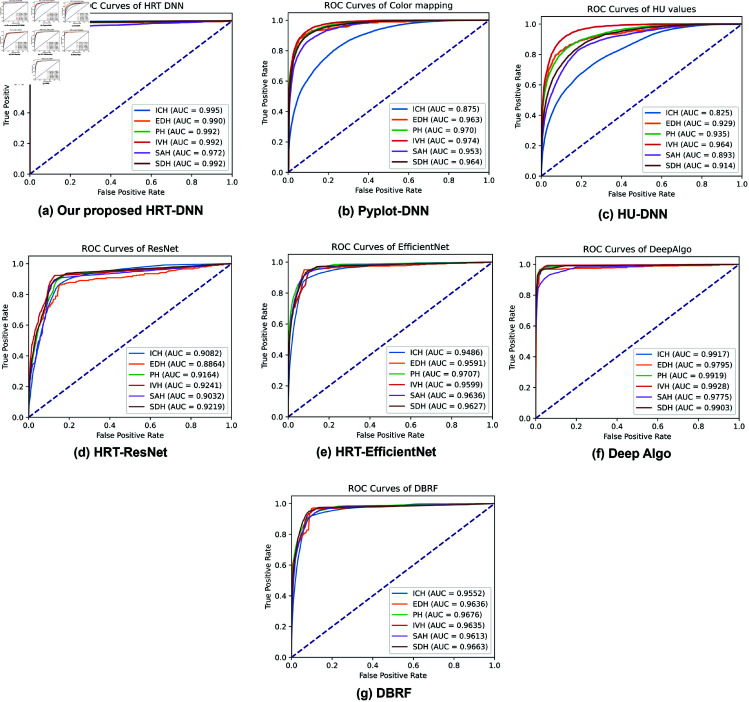
Comparison of ROC of multi-class classification models using RSNA dataset.

For each type of ICH, the results are reported in Table 4. With our HRT-DNN, the type PH has the highest sensitivity of 95.19% and the lowest is SAH of 84.46%. The other types of EDH, SDH, and IVS are 89.06%, 93.66%, and 91.02%. Compared to Deep Algo, the highest sensitivity is SDH with 94.46% and the lowest is PH with 84.46%; while the others are 88.02% for EDH, 89.54% for SAH, and 92.89% for IVH. The ROC curve of each model can be found in Fig 12. The ROC of our HRT-DNN shown in Fig 12(a) is similar to Deep Algo in Fig 12(f).

**Table 4:** Classification performance of each ICH type using RSNA dataset.

Method	Measure	EDH	SDH	SAH	PH	IVH
HRT-DNN	Acc.	99.94%	99.46%	99.25%	99.63%	99.69%
	Sens.	89.06%	93.66%	84.46%	95.19%	91.02%
	Spec.	100.00%	99.83%	99.95%	99.84%	99.97%
Pyplt-DNN	Acc.	99.42%	96.46%	96.69%	99.36%	97.78%
	Sens.	37.24%	57.58%	52.77%	75.80%	63.49%
	Spec.	99.72%	98.91%	98.77%	97.33%	98.88%
HU-DNN	Acc.	99.54%	95.18%	95.77%	96.74%	97.75%
	Sens.	26.04%	23.90%	8.19%	36.18%	36.12%
	Spec.	99.09%	99.68%	99.92%	99.61%	99.73%
HRT-ResNet	Acc.	85.76%	87.57%	90.57%	89.82%	88.86%
	Sens.	78.91%	82.55%	78.80%	80.72%	83.66%
	Spec.	85.80%	87.81%	90.95%	90.25%	89.18%
HRT-EfficientNet	Acc.	93.70%	93.44%	91.40%	94.71%	92.66%
	Sens.	81.77%	88.89%	85.28%	81.73%	88.78%
	Spec.	93.76%	93.66%	91.59%	95.32%	92.91%
Deep Algo [33]	Acc.	98.33%	98.47%	98.37%	98.16%	98.77%
	Sens.	88.02%	94.46%	89.54%	84.46%	92.89%
	Spec.	98.38%	98.66%	98.65%	98.81%	99.14%
DBRF [32]	Acc.	91.57%	92.67%	93.75%	92.96%	93.63%
	Sens.	84.64%	88.21%	83.23%	84.46%	89.42%
	Spec.	91.60%	92.88%	94.08%	93.36%	93.89%

#### 4.3.3 Classification performed by radiologist and resident.

#### Dataset.

A subset of 975 images were randomly selected from RSNA dataset, where 573 were ICH and 402 were non-ICH slices. Among the 573 ICH slices, the number of slices for each type which are EDH, IPH, IVH, SAH, and SDH were 57, 189, 151, 210, and 253, respectively. Each image was tested with the trained models, HRT-DNN and HU-DNN, and determined by a radiologist with 20 years of experience in neuroimaging and neuroradiology intervention and a resident with 2 years of experience in radiology training. The radiologist and resident were given DICOM files, where window parameters (WW and WL) can be adjusted to give the final diagnosis.

With the test of this dataset, HRT-DNN binary classification model in Table 5 achieved 97.91% sensitivity, 88.56% specificity, and 94.05% accuracy. The radiologist achieved, 98.60%, 90.05%, and 95.07%, while the resident achieved 97.73%, 91.04%, and 94.97%; respectively for sensitivity, specificity, and accuracy. HU-DNN obtained the lowest result of 46.60% sensitivity, 83.83% specificity, and 61.95% accuracy.

**Table 5:** Comparison of classification performance by an experienced radiologist, a resident, and our proposed method.

Label	Method (%)											
	HRT-DNN			HU-DNN			Radiologist			Resident		
	Acc.	Sens.	Spec.	Acc.	Sens.	Spec.	Acc.	Sens.	Spec.	Acc.	Sens.	Spec.
ICH/non-ICH	94.05%	97.91%	88.56%	61.95%	46.60%	83.83%	95.07%	98.60%	90.05%	94.97%	97.73%	91.04%
Five Types												
EDH	98.87%	84.21%	99.78%	94.36%	7.02%	99.78%	95.07%	98.24%	94.33%	94.76%	96.49%	94.66%
SDH	93.85%	93.68%	93.91%	80.51%	38.34%	95.29%	93.74%	93.67%	93.76%	94.25%	94.46%	94.18%
SAH	92.62%	76.67%	96.99%	80.21%	46.19%	89.54%	94.05%	92.38%	94.50%	94.46%	88.57%	96.07%
IPH	91.90%	96.83%	90.71%	86.15%	44.50%	95.93%	95.58%	96.29%	95.41%	93.84%	93.65%	93.89%
IVH	98.26%	95.36%	98.79%	86.36%	60.93%	91.02%	93.94%	94.70%	93.81%	94.97%	93.37%	95.26%
Avg.	95.10%	89.35%	96.03%	85.52%	39.04%	94.31%	94.38%	95.06%	94.37%	94.46%	93.31%	94.81%

For multi-class classification, HRT-DNN model achieved an average sensitivity of 89.35%, specificity of 96.03%, and accuracy of 95.10%. The SAH obtained the lowest sensitivity of 76.67%. The average sensitivity, specificity, and accuracy performed by the radiologist were 95.06%, 94.37%, and 94.38%, respectively. The lowest sensitivity was SAH type, however; the value was 92.38% for the radiologist and 76.67% for HRT-DNN. The resident achieved 93.31% sensitivity, 94.81% specificity, and 94.46% accuracy, whereas SAH obtained 88.57% sensitivity. The results of other types are shown in Table 5.

#### 4.3.4 Determining ICH types from preprocessed images.

The same dataset in Sect 4.3.3 was used in this experiment to compare the performance of the HU-RGB preprocessing methods when reviewing the scan by radiologists. The preprocessed slice with our HRT, Pyplot, DICOM files as in routine practice, and a mapping to intensity values with (40, 40) setting were reviewed by a radiologist and a resident for ICH type classification.

The average performance is listed in Table 6. The diagnosis with our HRT method achieved 96.72% sensitivity, 96.36% specificity, and 96.43% accuracy by the radiologist and 97.39%, 96.19%, and 96.43%, respectively by the resident. The performance using preprocessed images with Pyplot achieved 91.02% sensitivity, 88.34% specificity, and 88.84% accuracy by the radiologist and the resident obtained 89.89%, 88.99%, and 89.08%, respectively. Using the DICOM file as in the clinical routine, the radiologist achieved 95.06%, 94.37%, and 94.38%; while the resident achieved 93.31%, 94.81%, and 94.46% respectively for sensitivity, specificity, and accuracy. With a predefined windows parameter of WW=40 and WL=40; sensitivity, specificity, and accuracy were 90.36%, 88.90%, and 89.04%, respectively for the radiologist; and 90.13%, 88.64%, and 88.88% for the resident.

**Table 6:** Performance of determining ICH types by the radiologist and the resident with HRT, DICOM file, HU values mapped with (40,40).

	HRT			Pyplot			DICOM File			HU values (ww=40, wl=40)		
Reader	Acc.	Sens.	Spec.	Acc.	Sens.	Spec.	Acc.	Sens.	Spec.	Acc.	Sens.	Spec.
Radiologist	96.43%	96.72%	96.36%	88.84%	91.02%	88.34%	94.38%	95.06%	94.37%	89.04%	90.36%	88.90%
Resident	96.43%	97.39%	96.19%	89.08%	89.89%	88.99%	94.46%	93.31%	94.81%	88.88%	90.13%	88.64%

#### 4.3.5 BraTS dataset: Classification task on MRI.

We employed HRT method to preprocess by highlighting the tumor on MRI scans, followed by training with DenseNet121 with BraTS dataset. It was introduced for the Brain Tumor Segmentation (BraTS) challenge [41–43], utilizing multi-institutional pre-operative baseline multi-parametric magnetic resonance imaging (mpMRI) scans. We focused on classification tasks aimed at predicting the MGMT promoter methylation status.

Since the pixel values in the BraTS dataset come from MRI scans and do not correspond to Hounsfield Units (HU), mapping these values becomes essential for utilizing our HU-RGB conversion technique to delineate tumor components. We exclude mapped values greater than 1500 HU. Distinct predefined window settings were established for different volumes—T1-weighted (T1w), T1-weighted post-contrast (T1wCE), T2-weighted (T2w), and Fluid Attenuated Inversion Recovery (FLAIR)—each with unique settings. For T1w, T2w, and FLAIR, the window levels (WL) range from [1500, 200] to [2500, 3000], with intervals of 250 HU for each setting, while T1wCE employs intervals of 750 HU starting from [2500, 5000] to [6000, 9000]. The window width (WW) is set at 200 HU for T1w, T2w, and FLAIR, and 3000 HU for T1wCE.

**Fig 13:**
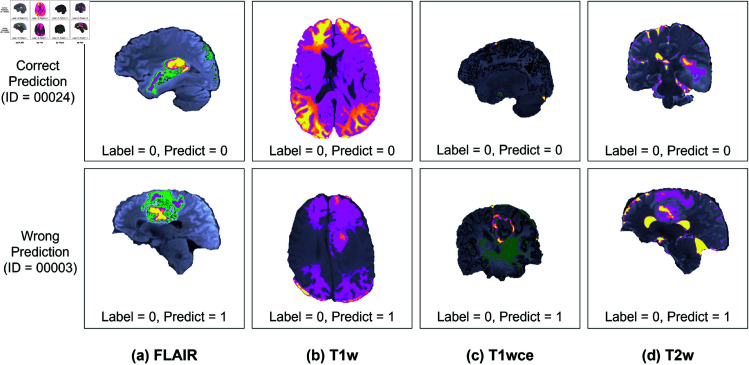
Examples of correct and incorrect classification result of each volume.

Table 7 presents the classification performance of tumor and normal slices. Our preprocessing method achieved an average accuracy of 80.95%, sensitivity of 74.99%, and specificity of 87.10%. The best result is obtained with the preprocessed FLAIR volume, achieving 85.71% accuracy, 81.25% sensitivity, and 90.32% specificity. Examples of HRT preprocessed for each type of volume and the prediction result is shown in Fig 13.

**Table 7:** Tumor classification of each slide from BraTS dataset using HRT method to preprocess the volume.

Volume	Tumor/Normal Classification		
	Acc. (%)	Sens. (%)	Spec. (%)
FLAIR	85.71%	81.25%	90.32%
T1w	73.01%	65.62%	80.64%
T1wCE	82.54%	75.00%	90.32%
T2w	82.54%	78.12%	87.10%
Average	80.95%	74.99%	87.10%

#### 4.3.6 Ablation study.

In this section, we study the impact of the predefined threshold values for locating ROI of hemorrhage, CSF, white-matter, and gray-matter in Eqs 2, (3), (4), and (5), respectively. We remove the condition of f(P(i,j)) that compares the HU of each selected pixel with the predefined threshold. The preprocessed brain CT images were trained with DenseNet121 with the same settings as the prior experiments to perform binary classification and multi-class classification. For a fair comparison, we evaluated the classification performance using the same set of training and testing images as in Table 1.

The results in Table 8 show that employing the predefined thresholds achieved higher accuracy, sensitivity, and specificity especially when testing with the CQ500 dataset. For the binary classification using HU-RGB images without threshold obtained 87.63% accuracy, 83.90% recall, and 92.83% specificity; while the ones with threshold achieved 92.67%, 89.31%, and 97.35%; respectively.

**Table 8:** Classification result using the proposed HU-RGB with and without the predefined thresholds for location ROI of each brain’s component.

Dataset	Label	Classification Result using HRT-DNN (%)					
		HU-RGB with Threshold			HU-RGB without Threshold		
		Acc.	Sens.	Spec.	Acc.	Sens.	Spec.
RSNA	ICH/non-ICH	98.40%	95.09%	98.93%	98.15%	94.47%	98.73%
	Five Types						
	EDH	99.94%	89.06%	99.99%	99.94%	89.06%	99.99%
	SDH	99.46%	93.66%	99.82%	99.41%	93.13%	99.81%
	SAH	99.25%	84.46%	99.95%	99.20%	83%59	99.94%
	PH	99.63%	95.19%	99.84%	99.58%	94.54%	99.81%
	IVH	99.69%	91.02%	99.96%	99.67%	90.65%	99.96%
	Average	99.59%	90.68%	99.91%	99.56%	90.19%	99.90%
CQ500	ICH/non-ICH	92.67%	89.31%	97.35%	87.63%	83.90%	92.83%
	Five Types						
	EDH	98.97%	63.64%	99.60%	95.98%	63.64%	96.55%
	SDH	95.59%	91.96%	95.94%	92.67%	88.39%	93.08%
	SAH	96.45%	84.09%	97.89%	94.17%	81.82%	95.60%
	PH	91.80%	81.79%	95.89%	88.81%	77.99%	93.23%
	IVH	95.98%	79.73%	96.99%	92.20%	72.97%	93.39%
	Average	95.76%	80.24%	97.26%	92.76%	76.96%	94.37%

When investigating each ICH type, we found that the preprocessed images using our HRT with the threshold values have higher sensitivity values for four types except the EDH type. This is because the bleeding in EDH occurred in the outermost of the brain, so employing a threshold of 110 in Eq 2 to remove any calcium in the brain tissue has no effect.

## 5 Discussion

In this study, we discovered that selecting an appropriate window setting for mapping HU to RGB color components can be accomplished through the utilization of boundary points obtained from the contour of region extracted using multiple predefined window settings and various brain components. Our findings indicate that the binary model trained with our HRT preprocessed images outperforms both the HU-RGB mapping with the Pyplot library and the grayscale image mapped with the settings WW=40 and WL=40.

Our proposed classification model (HRT-DNN), trained with the RSNA dataset, achieved remarkable performance metrics, including an accuracy, sensitivity, and specificity of 98.40%, 95.10%, and 98.93% respectively, with an AUC of 0.9978 for binary classification. For multi-class classification, the model attained 99.59% accuracy, 91.48% sensitivity, and 99.92% specificity, with an AUC of 0.9994. Compared to prior works utilizing stacked preprocessed brain CT with multiple predefined windows such as Deep Algo [33] and DBRF [32], our HRT-DNN exhibited superior performance. However, it is noteworthy that the performance of HRT preprocessed images with ResNet50 and EfficientNet-B0 architectures was comparatively lower. In comparison with expert radiologists, our HRT-DNN model demonstrated classification performance comparable to that of a 20-year-experienced radiologist and even surpassed that of a 2-year-experienced resident. Experimental results revealed that the sensitivity of identifying subarachnoid hemorrhage (SAH) by the resident was 88.57%, whereas the radiologist achieved 92.38%.

In clinical routines, radiologists adjust windowing parameters in DICOM files to enhance visibility. Preprocessing brain CT scans with HRT, by aligning bleeding patterns, significantly enhances radiologists’ reading effectiveness. This process aids in the identification of distinct visual cues, enabling a more accurate diagnosis and differentiation of various ICH types. HRT streamlines the diagnostic process, facilitating a quicker and more precise recognition of the specific characteristics that define different ICH categories. Both radiologists and residents exhibited improved classification of the five intracranial hemorrhage (ICH) types compared to DICOM files. Specifically, sensitivity and specificity in identifying SAH by the radiologist were 95.71% and 95.94%, respectively, and by the resident were 96.66% and 96.86%, respectively, representing improvements over the DICOM-based approach.

To demonstrate the generalizability of our proposed method, we conducted external validation using the CQ500 dataset. The HRT-DNN showcased significantly higher results with an AUC of 0.9778, in contrast to Pyplot and a fixed window transformation, which obtained AUC values of 0.7291 and 0.4811, respectively.

Despite the promising results, our proposed brain CT preprocessing method has certain limitations. The selection of suitable CT window settings in HRT is based on predefined settings optimized for the scanners used in our datasets and specific regions of interest, primarily focusing on scanners employed in the RSNA dataset and hemorrhagic stroke bleeding. This may not be universally applicable across different scanners or patient populations without further adjustment. Nonetheless, our experiments on the CQ500 dataset and tumor classification in MRI (BraTS dataset) suggest potential for adaptation. Additionally, performance may be influenced by the quality of non-contrast CT images, including resolution, contrast, and the presence of artifacts, which can vary significantly across clinical settings.

## 6 Conclusion

We introduce a novel method termed HU-RGB transformation with multiple windows and multiple components (HRT), which leverages predefined window settings to display various brain components such as hemorrhage stages, CSF, and white-and-gray matter. Each predefined setting is coupled with a Sigmoid function to map HU to RGB color components, facilitating the identification of potential regions of interest. The selection of suitable windowing parameters for visualizing intracranial hemorrhage involves utilizing the detected brain components to eliminate extraneous areas such as calcium in the brain and skull. In the Hounsfield unit (HU) scale, low HU represents air or liquid, and high HU indicates soft tissue to calcium. By comparing the number of boundary points extracted from the region of the mapped images from low to high HU, we determine the (WW, WL) value that induces significant decreases due to the lower HU values and larger area of liquid in the brain.

The proposed HRT method serves as an image preprocessing technique aimed at enhancing the performance of intracranial hemorrhage (ICH) classification and aiding radiologists in their inspections. Our experimental results demonstrate that images preprocessed using HRT and trained with deep neural networks for binary or multi-class classification of the five types of ICH yield superior outcomes. Furthermore, these preprocessed images can be readily utilized by both radiologists and residents during diagnosis, assisting in the accurate determination of the ICH type.

In future research, we aim to explore different image modalities for creating RGB mapped images to improve classification performance by machine learning model. We also target deep neural network architectures and employ ensemble methods to further enhance the performance of multi-class classification.
